# Distinct Neural Correlates Underlie Inhibitory Mechanisms of Motor Inhibition and Motor Imagery Restraint

**DOI:** 10.3389/fnbeh.2020.00077

**Published:** 2020-06-03

**Authors:** Peter E. Yoo, Thomas J. Oxley, Maureen A. Hagan, Sam John, Stephen M. Ronayne, Gil S. Rind, Alexander M. Brinded, Nicholas L. Opie, Bradford A. Moffat, Yan T. Wong

**Affiliations:** ^1^Vascular Bionics Laboratory, Department of Medicine, Royal Melbourne Hospital, The University of Melbourne, Parkville, VIC, Australia; ^2^Department of Electrical & Electronic Engineering, The University of Melbourne, Parkville, VIC, Australia; ^3^The Florey Institute of Neuroscience and Mental Health, Parkville, VIC, Australia; ^4^Department of Physiology, Monash University, Melbourne, VIC, Australia; ^5^St. Vincent’s Hospital, Melbourne, VIC, Australia; ^6^Department of Anatomy and Neuroscience, The University of Melbourne, Kenneth Myer Building, Parkville, VIC, Australia; ^7^Department of Electrical and Computer Systems Engineering, Monash University, Melbourne, VIC, Australia; ^8^Neuroscience Program, Biomedicine Discovery Institute, Monash University, Melbourne, VIC, Australia

**Keywords:** fMRI, motor, inhibition, motor network, motor imagery

## Abstract

There is evidence to suggest that motor execution and motor imagery both involve planning and execution of the same motor plan, however, in the latter the output is inhibited. Currently, little is known about the underlying neural mechanisms of motor output inhibition during motor imagery. Uncovering the distinctive characteristics of motor imagery may help us better understand how we abstract complex thoughts and acquire new motor skills. The current study aimed to dissociate the cognitive processes involved in two distinct inhibitory mechanisms of motor inhibition and motor imagery restraint. Eleven healthy participants engaged in an imagined GO/NO-GO task during a 7 Tesla fMRI experiment. Participants planned a specific type of motor imagery, then, imagined the movements during the GO condition and restrained from making a response during the NO-GO condition. The results revealed that specific sub-regions of the supplementary motor cortex (SMC) and the primary motor cortex (M1) were recruited during the imagination of specific movements and information flowed from the SMC to the M1. Such condition-specific recruitment was not observed when motor imagery was restrained. Instead, general recruitment of the posterior parietal cortex (PPC) was observed, while the BOLD activity in the SMC and the M1 decreased below the baseline at the same time. Information flowed from the PPC to the SMC, and recurrently between the M1 and the SMC, and the M1 and the PPC. These results suggest that motor imagery involves task-specific motor output inhibition partly imposed by the SMC to the M1, while the PPC globally inhibits motor plans before they are passed on for execution during the restraint of responses.

## Introduction

The neural correlates of voluntary movement (i.e., *motor execution*) and the imagination of the same movements (i.e., *motor imagery*) overlap extensively (Jeannerod, 1995; Decety, 1996a; Hotz-Boendermaker et al., 2008). Fittingly, studies suggest that motor execution and motor imagery involve planning of the same motor program; however, in the latter, the output is inhibited (Jeannerod, 1995; Decety, 1996b; Munzert et al., 2009). The performance of motor imagery is constrained by the physical limitations associated with the congruent movements (e.g., the time taken to imagine performing a certain task is the same as actually performing the task; Guillot and Collet, 2005; Dahm and Rieger, 2016), the imagination of upper and lower limbs increase corticospinal excitability (Fadiga et al., 1999; Grosprêtre et al., 2016) and somatotopic cortical activations (Lotze et al., 1999; Buccino et al., 2001; Ehrsson et al., 2003; Solodkin et al., 2004; Guillot and Collet, 2008), and congruent patterns of sub-threshold electromyographic (EMG) activity has been observed during imagination of specific movements (Gandevia et al., 1997; Hashimoto and Rothwell, 1999). However, the exact neural mechanism underlying motor imagery is still a widely debated topic due to the dichotomy of overlapping neural correlates, and the obvious cognitive and physical differences across the two motor processes (Guillot et al., 2012; Criaud and Boulinguez, 2013). Uncovering the characteristic features of motor imagery may help us better understand how we acquire new motor skills and evolved to perform complex abstraction.

If indeed motor execution and motor imagery both involve planning of the same motor program, we hypothesize that the defining characteristic feature of motor imagery is a task-specific inhibition of the motor output. Previously, motor output inhibition has been investigated using a GO/NO-GO paradigm, where the participants plan for a specific movement, then either execute or restrain from making the response upon a “GO” or “NO-GO” cue, respectively (Simmonds et al., 2008). However, the inhibitory mechanisms underlying the motor imagery (i.e., GO) and NO-GO condition are cognitively different. During the former, the actor does not know to inhibit the motor output until the NO-GO cue is given. Furthermore, the latter task can be achieved by general response restraint (i.e., motor restraint), while the actor does not have to perform other actions or cognitions simultaneously, unlike in the case of motor imagery (i.e., inhibiting and imagining the movement at the same time).

Considering the above, we attempted to delineate the cortical networks of two distinct inhibitory mechanisms, *motor inhibition* and *motor imagery restraint*. In this study, motor inhibition refers to the inhibition of movements during the imagery (i.e., GO condition), motor imagery restraint refers to the act of restraining from imagining the movements (i.e., NO-GO condition) and motor planning refers to the planning of the motor imagery. Participants engaged in an imagined GO/NO-GO motor task during a 7-tesla functional magnetic resonance imaging (7T-fMRI) experiment. The neural correlates of motor inhibition and motor imagery restraint were dissociated by contrasting the blood-oxygenation-level-dependent (BOLD) states yielded between the GO and NO-GO conditions against each other. The preferentiality and the spatiotemporal dynamics of significant BOLD activation were quantified to investigate the neural mechanisms underlying the inhibitory processes.

## Materials and Methods

### Participants

Eleven healthy volunteers (five males and six females; mean ± standard deviation age: 25 ± 5 years) participated in a single-session fMRI experiment after giving informed consent (The University of Melbourne Human Ethics Committee approved this study; Ethics ID: 1340926.1).

### Behavioral Protocol

**Figure 1:**
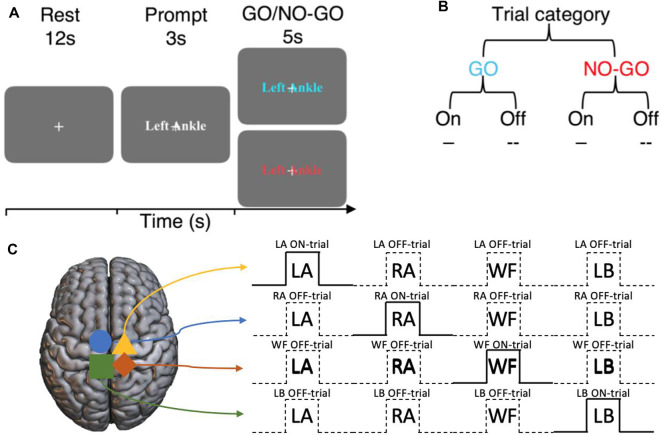
Experimental design of the imagined GO/NO-GO task and the terminology used to describe trial types. **(A)** Each trial consists of a 12 s rest block, a 3 s prompt block then a 5 s GO or NO-GO trial. **(B)** Each trial can either be a GO or a NO-GO trial (75:25 ratio). For a given set of significant voxels in a particular condition, the corresponding trial blocks of that condition is referred to as ON-trials, and the rest are referred to as OFF-trials. **(C)** An example of ON- and OFF-trials for the significant voxels for each condition.

The participants engaged in an imagined GO/NO-GO task by following the instructions presented on the screen (Figure 1A). An experimental trial lasted for 20 s, consisting of a 12 s rest block, a 3 s prompt block, then a 5 s GO/NO-GO block. The movement to be imagined was presented during the prompt blocks in random order. There were four motor imagery conditions, “Left Ankle” (LA), “Right Ankle” (RA), “Walk Forward” (WF), and “Lean Back” (LB). Well-defined (e.g., LA and RA), as well as more abstract movements (e.g., WF and LB) were chosen, to investigate if the potential inhibitory mechanisms extend across a variety of movement types and complexity. During the GO trials, the participants imagined the corresponding movements. During the NO-GO trials, the participants were to refrain from performing the motor imagery and resort back to rest. Each GO trial condition was repeated six times, and NO-GO trial was repeated 2 times (prevalence ratio of GO:NO-GO trials, 75:25; total trials across participants = 352). All experiments finished with 12 s of rest block. The entire task lasted for 10 min and 57 s. Participants were instructed to imagine performing the movements by concentrating on the feeling of the movements and to visualize the movements if desired, with their eyes fixated on a center cross at all times. It was to imagine in a first-person point-of-view and to keep completely still. The presence of overt movements was monitored visually from the MRI console desk.

### Terminology

For the set of sub-regions showing a significant BOLD activation during a specific motor imagery condition, *ON-trials* refer to the time-points when the corresponding movements were imagined. Within the same sub-region, *Off-trials* refer to the time-points when all other movements were imagined (Figure 1B). That is, ON and OFF-trials refer to specific time-points during the experiment within the same set of BOLD activation regions. The regions are defined by the motor imagery condition that the BOLD activation corresponds to. For example, consider the sub-region that shows a significant BOLD activation during the left ankle imagery (i.e., LA GO > NO-GO). For this sub-region, the time-points when left ankle movements were imagined are the ON-trials, and the overall spatiotemporal component is together referred to as LA ON-trials. On the other hand, for the same sub-region, the time-points when the movements of right ankle, walk forward, and lean back were imagined are the OFF-trials and the overall spatiotemporal component is together referred to as LA OFF-trials, and so on (Figure 1C).

### fMRI Protocol

#### Image Acquisition

All imaging was performed on a 7T research scanner (Siemens Healthcare, Erlangen, Germany) with a 32-channel head-coil (Nova Medical Inc., Wilmington MA, USA). A T_1_-weighted structural image was acquired for each participant using a magnetization-prepared rapid gradient-echo sequence (MP2RAGE; voxel volume = 0.9 × 0.9 × 0.9 mm^3^; iPAT factor = 4; TR = 4,900 ms; transmitter voltage = 240V).

All fMRI images were acquired using 2D gradient echo-echo planar imaging (GE-EPI) with multiband and parallel imaging acceleration [Siemens Healthcare prototype research sequence; bandwidth = 1,690 Hz/pixel; echo time = 24 ms; repetition time = 500 ms; echo spacing = 0.74 ms; EPI factor = 148; phase encoding shift factor = 2; voxel volume = 1.5 × 1.5 × 1.5 mm^3^; in-plane field of view (FOV) = 222 × 222 mm^2^; flip angle = 34°, where T_1_ = 2,000 ms; partial Fourier = 6/8; phase encoding direction = A-P; multiband factor = 3; GRAPPA factor = 3; number of slices = 27; slice FOV = 40.5 mm]. Two additional sets of GE-EPI images with opposing phase encoding directions were acquired to perform B_0_-distortion correction.

#### Image Analysis

A customized analysis pipeline, optimized partial-coverage functional analysis pipeline (OPFAP; Yoo et al., 2018a), was used due to the partial-coverage 7T-fMRI data. We recommend referring to our previous work for specific technical information (Yoo et al., 2018a).

The FMRIB’s Software Library’s (FSL v6.0) was used for functional analyses (Jenkinson et al., 2012). Susceptibility-induced off-resonance field was corrected using the opposite phase-encoding direction GE-EPI images *via* FSL’s *top-up* and *applytopup* functions (Jenkinson et al., 2012). Then, the distortion-corrected functional images were motion corrected, high-pass filtered (0.01 Hz), skull-stripped, but were not smoothed. No slice-timing corrections were employed given the fast TRs and multiband acceleration.

The functional images were subjected to general linear model analysis. The prompt, GO and NO-GO blocks were modeled as separate box-car functions, convoluted with a gamma function (mean = 6 s, standard deviation = 3 s). The time-points of significant movement artifacts identified during the motion-correction step were included in the model as nuisance variables to further control for the motion artifacts. The resulting *Z*-score maps of the GO trials were contrasted against the corresponding NO-GO trials (e.g., LA GO>LA NO-GO). Significant activation was defined using a lower *Z*-score threshold of 2.3 (with *p* < 0.05 for significance testing; cluster-based correction).

The individual-level activation maps were masked with the participant-specific region of interest (ROI) masks to ensure fair comparisons of metrics within the dorsal motor network regions across participants. The ROIs were manually defined in a study-specific template brain space, then were transformed into each participant’s functional space. The ROI masks were created for the posterior parietal cortex (PPC), the supplementary motor cortex (SMC), and the primary motor cortex (M1) using the following steps. First, a study-specific template brain was created using all T1-weighted anatomical images using the Advanced Normalization Tools (ANTs; Avants et al., 2011). Note that during the creation of the study-specific template brain, the affine transformation and deformation parameters from template brain space to each individual’s anatomical space were calculated and saved (i.e., anatomical space ←→ template space). Second, each participant’s center time-point of the fMRI image series (i.e., motion-correction reference image) was linearly registered to the corresponding T1-weighted anatomical images using FSL’s LInear Registration Tool (FLIRT) with boundary-based registration to calculate the transformation parameters from the functional space to the anatomical space (i.e., functional space → anatomical space). Third, the masks were manually drawn slice-by-slice axially, for the PPC, the SMC, and the M1 in the study-specific template brain. Fourth, using the inverse of the transformation and deformation parameters calculated above, these masks were transformed into the participants’ own functional image spaces in a step-wise manner [i.e., template space → anatomical space (*via* ANTs) → functional space (*via* FLIRT)]. In turn, three ROI masks were created for each participant.

A group-level analysis was also performed. The contrast of parameter estimates and the variance maps from the individual-level analysis above were linearly registered into each subject’s T_1_-weighted anatomical space using boundary-based registration. The resulting images were non-linearly registered into the study-specific template space using ANTs using a mutual information cost function. Using the resulting images, a group-level statistical test was carried out in FSL. Significant activation was defined using a lower *Z*-score threshold of 2.3 (with *p* < 0.05 for significance testing; cluster-based correction).

### Statistics

In any case of multiple comparisons, the significance of the *p*-values was tested against the False-Discovery Rate (FDR) adjusted threshold using the Benjamini–Hochberg procedure at *Q* = 0.05. For the tests that have survived the FDR-adjusted threshold, the original *p*-values were reported in the text.

### Preferentiality Testing of Motor Inhibition and Restraint BOLD Activity

We quantified the preferentiality of BOLD activity during specific motor imagery conditions (e.g., GO blocks) to investigate the neural correlates of motor inhibition and restraint. First, the BOLD percent signal change (%Δ*S*) was calculated by normalizing the data by subtracting the temporal mean signal then dividing it by the temporal mean signal of the voxel at each time point.

To investigate the neural correlates of motor inhibition, the %Δ*S* time-courses were extracted from the voxels showing significant activation during LA, RA, WF, and LB GO blocks within the PPC, the SMC, and the M1. The extracted time-courses were then averaged across voxels per condition and per region. The %Δ*S* values between 4 s and 9 s after the start of the ON-trial GO blocks were compared against each of the three OFF-trials across the participants within each region using Wilcoxon rank-sum tests (no trial averaging). To provide further support of the preferentiality of BOLD activity, random combinations of 6 ON and OFF-trials were sampled across all participants’ voxel averaged data for each region and condition. Then, all the ON and OFF-trial data were compared against each other without averaging across the samples within each region and condition using Wilcoxon rank-sum tests. This process was permuted 10,000 times with no repeating combinations of trials. The average percentage of significant predictive activity (i.e., ON-trials>OFF-trials) was calculated across permutations for each region and condition.

To investigate the neural correlates of motor imagery restraint, the lack of condition-specific BOLD predictability was tested, because the group-level significant activation was observed in general across conditions and not for specific conditions. The %Δ*S* time-courses were extracted from voxels showing a significant activation during all NO-GO blocks at the individual level per ROI. The average %Δ*S* values between 4 s and 9 s after the start of the ON-trial GO blocks were compared against each of the three OFF-trials across the participants within each region using Wilcoxon rank-sum tests (no trial averaging).

### Spatiotemporal Dynamic Analysis of BOLD Activity During Motor Inhibition and Restraint

Then, we investigated the spatiotemporal dynamics of BOLD activation and deactivation onset. We controlled for any region-specific hemodynamic response function (HRF) driving the potential differences in the spatiotemporal dynamics across the dorsal motor network. We estimated the HRF of each voxel using a blind approach, then deconvoluted the BOLD signal with the estimated HRF (Wu et al., 2013). We compared the BOLD activation time-courses across the ROI masks. In regions of significant activation, the latency to reach the peak %Δ*S* was compared against each other across participants using Wilcoxon rank-sum tests. In regions of significant deactivation, the latency to reach the trough %Δ*S* was compared.

We further investigated how the information flow changed across the different ROIs during motor inhibition and motor imagery restraint. We subjected the voxel and trial averaged %ΔS time-courses from each region to a Multivariate Granger Causality (MVGC) analysis (Barnett and Seth, 2014). The ON and OFF-trials were separately averaged across trials. There were three variables (PPC, SMC, and M1) and 11 observations (one for each subject). The sample rate was set at 2 Hz (i.e., 500 ms) to match the TR used in fMRI acquisition. The maximum model order was set at 10 as lags greater than 5 s were not expected between the regions considering that the GO/NO-GO blocks lasted for 5 s. Granger’s *F*-tests were calculated to compare the G-causality between the regions. The actual model order was used for statistical testing, which in this case was seven volumes (3.5 s). Significance was defined at *p* < 0.05 with FDR multiple comparisons correction.

## Results

### Regions of the Dorsal Motor Network Show BOLD Activation During Planning, Imagination, and Restraint of Motor Imagery

**Figure 2:**
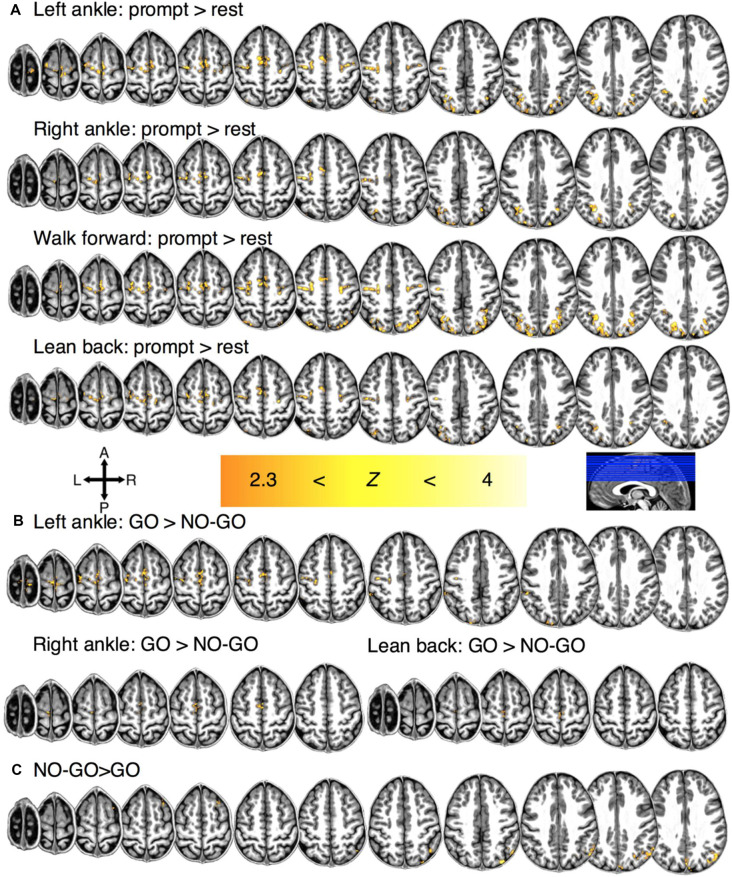
Regions of significant group-level BOLD activation for **(A)** motor planning for each condition. **(B)** Regions of significant activation during motor imagery for each condition and **(C)** motor restraint in general.

To establish the network involved in motor planning, motor imagery, and motor imagery restraint, we first identified the regions that showed significant BOLD activation during prompt, GO and NO-GO conditions, respectively (Supplementary Table S1). Significant BOLD activation was observed during motor planning at the individual-level in the PPC (*Z* > 2.3, *p* < 0.05, cluster-wise corrected; participant and condition mean VV ± SE; 5,838 ± 697 mm^3^) and the SMC (1,402 ± 215 mm^3^) for all participants. Significant group-level activation was observed bilaterally for each condition in the PPC (condition mean VV ± SE; 2,190 ± 919 mm^3^) and the SMC (1,091 ± 206 mm^3^; Figure 2A).

In 8/11 participants, significant BOLD activation was observed during motor imagery (e.g., LA GO>LA NO-GO blocks) at the individual-level in the M1 (351 ± 76 mm^3^). In the 9/11 participants, significant activation was also observed in the SMC (776 ± 123 mm^3^) and the PPC (2,872 ± 462 mm^3^). Significant group-level activation was observed in the SMC bilaterally and in the M1 contralaterally for conditions LA (932 mm^3^), RA (240 mm^3^), and LB (84 mm^3^) but not for WF (Figure 2B).

Lastly, a significant individual-level BOLD activation was observed in the PPC during motor imagery restraint across all conditions in general for all participants (5,715 ± 688 mm^3^; i.e., NO-GO>GO blocks). Significant BOLD activation was observed at the group-level across all conditions in general in the right PPC (1,048 mm^3^; Figure 2C). There was no condition-specific activation during imagined movement restraint.

### The SMC Activates During Motor Inhibition, While the PPC Activates During Motor Imagery Restraint

**Figure 3:**
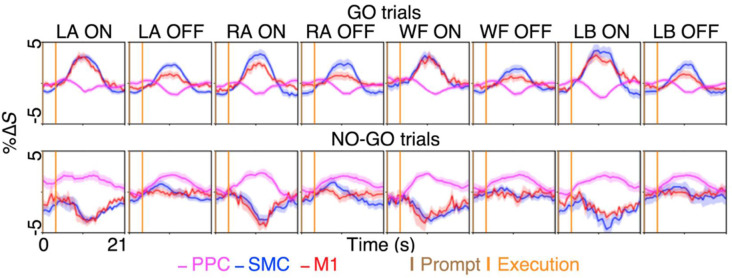
The BOLD activation time-course during motor imagery and restraint across the dorsal motor network areas. A preferential BOLD activity was observed during the imagination of left ankle (LA), right ankle (RA), walk forward (WF) and lean back (LB), in spatially distinct sub-regions within the supplementary motor cortex (SMC) and the primary motor cortex (M1). The %Δ*S* during the ON-trial was significantly greater than %Δ*S* during the OFF-trials during the GO blocks for both the SMC and the M1 across the conditions (all *p* < 0.0001). Interestingly, selective BOLD deactivation was observed in the same sub-region of the SMC and the M1 during the NO-GO blocks for each condition, while general BOLD activation in the posterior parietal cortex (PPC) across the conditions coincided with the deactivations. The figures plot the voxel and trial mean %Δ*S* averaged across participants. The magenta curves plot the BOLD %Δ*S* time-courses at different times, from the voxels activating significantly during motor restraint within the PPC (i.e., NO-GO>GO). The blue and red curves plot the BOLD %Δ*S* time-course from the voxels activating significantly during specific motor imagery within the SMC and the M1, respectively (i.e., LA GO>LA NO-GO). The shading surrounding the curves depict the standard errors across participants. The brown and orange up-right bars indicate the start of prompt and execution blocks, respectively.

We investigated the cortical regions involved in motor inhibition and motor imagery restraint by quantifying the preferentiality of the BOLD activation during the GO and NO-GO blocks. The results revealed that during motor imagery, and thus motor inhibition, different sub-regions of the SMC and the M1 showed preferential BOLD activation to specific imagery conditions. Averaging across significant voxels of activation revealed greater %Δ*S* during the ON-trials compared to the OFF-trials for each condition in the SMC and the M1 (all *p* < 0.0001; Figure 3; Supplementary Table S2). To further validate the preferentiality of BOLD activation, we compared the voxel-averaged %Δ*S* of six randomly sampled ON and OFF-trials across all participants within each condition and ROI. On average across 10,000 permutations with no repeats, preferential BOLD activations were observed for the conditions, LA, RA, WF, and LB: 74%, 65%, 72% and 82% of the time in the SMC; and 83%, 70%, 86% and 95% of the time in the M1 (all *p* ≤ 0.0333).

In contrast, the results revealed that during motor imagery restraint (i.e., NO-GO blocks), there was no significant group-level BOLD activation in the SMC or the M1. Instead, the BOLD activation was observed primarily in the PPC and was not preferential for specific motor imagery conditions (Figure 2C). Averaging across voxels showing BOLD activation in the PPC revealed no significant differences in %Δ*S* between the ON-trials and OFF-trials during motor imagery restraint (all *p* > 0.0500; Figure 3). Interestingly, a significant decrease in %Δ*S* during the ON-trials compared to the OFF-trials was observed during motor imagery restraint in the same areas of the SMC and the M1 that were activating during motor inhibition (all *p* < 0.0001).

### The Spatiotemporal Dynamics of BOLD Activity Is Distinct During Motor Inhibition and Motor Imagery Restraint

**Figure 4:**
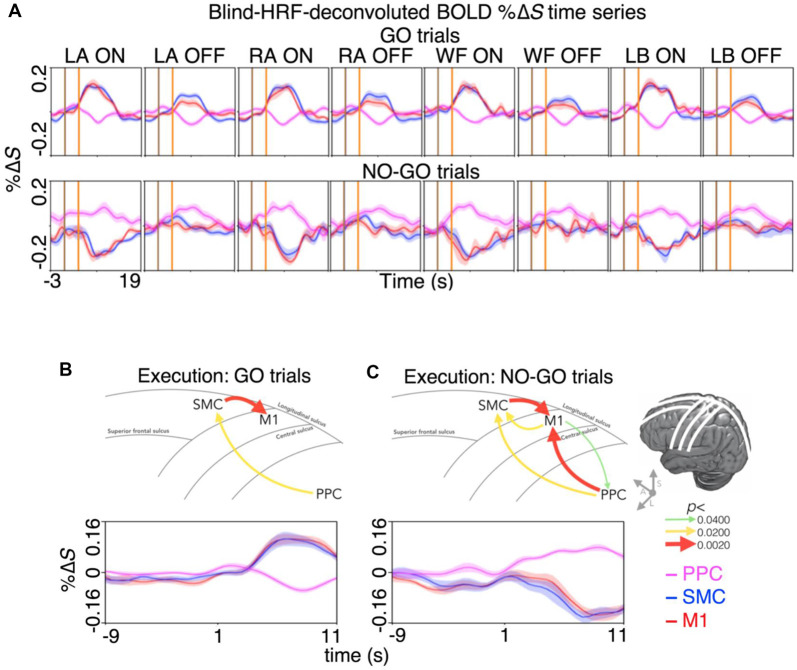
Spatiotemporal dynamics of BOLD activity and directional information flow across the dorsal motor network during motor inhibition and motor restraint. **(A)** The condition and region-specific trial-averaged BOLD %Δ*S* time-courses during motor inhibition (top row) and motor restraint (bottom row). During motor inhibition, BOLD activity peaked at the same time in the M1 and the SMC. During motor restraint, BOLD activity in the SMC and the M1 troughed at the same time as BOLD activity peaked in the PPC. The pattern of recurrent information flow across the dorsal motor network differed during **(B)** motor inhibition and **(C)** motor restraint. Averaging across the significant voxels and trials revealed a G-causal relationship from the SMC to the M1 during motor inhibition. During motor restraint, G-causal relationships were revealed from the PPC to the SMC and the M1. The line drawing and the arrows depict the major sulci of the brain and the direction of G-causal relationships. The curves plot the voxel, trial and participant averaged %Δ*S* of each region.

Lastly, we quantified the spatiotemporal dynamics of BOLD activity across the areas involved in motor inhibition and motor imagery restraint. During motor inhibition, the BOLD activation in the SMC appeared at the same time as in the M1, where there was no significant difference between the latency to the peak %Δ*S* from the start of the prompt blocks (mean ± SE; both the SMC and the M1 7.5 ± 0.5 s; *p* > 0.0500; Figure 4A). During motor imagery restraint, the BOLD deactivation in the SMC and the M1 appeared at the same time as the activation in the PPC. There was no significant difference between the latency to the trough %Δ*S* in the SMC (9.0 ± 0.4 s) and the M1 (8.5 ± 0.6 s), and the latency to reach the peak %Δ*S* in the PPC (6.9 ± 1.0 s; all *p* > 0.0500; Figure 4A).

Furthermore, the %Δ*S* time-courses were subjected to an effective connectivity analysis to investigate the directional information flow. Averaging across the significant voxels and trials revealed G-causal relationships from the SMC to the M1 (*p* < 0.0001) and from the PPC to the SMC during motor inhibition (*p* = 0.0149; Figure 4B). During motor imagery restraint, the G-causal relationships were observed from the PPC to the M1 (*p* = 0.0040), from the M1 to the PPC (*p* = 0.0388) and from the M1 to the SMC (*p* = 0.0071) and that from the SMC to the M1 (*p* < 0.0001) and from the PPC to the SMC remained (*p* = 0.0133; Figure 4C).

## Discussion

We distinguished the neural correlates of motor inhibition and motor imagery restraint using an imagined GO/NO-GO task. During motor inhibition, different spatial patterns of preferential BOLD activity were observed primarily in the SMC and the M1 for each type of imagined movement (Figures 2B, 3). BOLD activation appeared at the same time in the SMC and the M1 (Figure 3), and information flowed from the SMC to the M1 (Figure 4A). In contrast, no condition-specific BOLD activation was observed during motor imagery restraint. However, significant BOLD activation was observed in the PPC in general (Figure 2C) with simultaneous BOLD deactivation in the SMC and the M1 (Figure 3). Furthermore, information flowed from the PPC to the SMC, and recurrently between the M1 and the SMC, and the M1 and the PPC (Figure 4B). These results suggest that the SMC inhibits the outputs of specific motor plans during motor imagery while the PPC may globally inhibit motor plans before they are passed on for execution during motor imagery restraint.

The current inhibitory network overlaps well with previous motor execution studies (Coxon et al., 2006; Mattia et al., 2012; Osada et al., 2019), which provides support that congruent motor programs are planned during motor imagery. The inhibitory influence of the SMC on the M1 during motor imagery is also consistent with previous work. The SMC has inter-hemispherical connections and is highly interconnected with the PPC (Jones and Powell, 1970; Jürgens, 1984; Petrides and Pandya, 1984; Cavada and Goldman-Rakic, 1989), the M1, and the spinal cord (Cunnington et al., 2005). Kasess et al. (2008) showed that the human SMC actively suppresses the M1 during motor imagery tasks, where a weaker BOLD intensity was observed during motor imagery compared to motor execution. They further showed using a dynamic causal model that direct projection of inhibitory process to the SMC and a modulatory input from the SMC to the M1 was the best performing model to predict the observed preferential BOLD activity.

The current study extends upon previous findings and shows that the inhibitory mechanism of the SMC on the M1 is also task-specific. Despite a degree of overlap, spatially distinct patterns of BOLD activity were observed during the imagination of specific movements. Furthermore, the BOLD activation within each distinct pattern was preferential for each movement. Previous decision-making and executive-function studies have shown that SMC is involved in complex motor response tasks that require inhibition of distracting stimuli (Curtis et al., 2005; Nachev et al., 2005; Aron and Poldrack, 2006; Li et al., 2006; Sumner et al., 2007) or changes in motor goals in reaction to incoming information that require inhibition of previously intended motor output (Ullsperger and von Cramon, 2001; Ridderinkhof et al., 2004; Nachev et al., 2005). To our knowledge, the task-specific inhibitory mechanism of the SMC during motor imagery has not been demonstrated in humans.

A task-specific inhibitory mechanism is self-evident, as one can still perform overt actions while simultaneously imagining alternate tasks or planning for the next move—a behavior that requires inhibition of specific movements while performing others. Current results are also consistent with our previous work, where it was shown that specific sub-regions of the SMC orchestrates the flow of cortical information across the dorsal motor network to encode and execute specific motor plans (Yoo et al., 2018b). It is unlikely that these specific sub-regions of activity can be attributed to motor planning in this study, as the motor planning was experimentally separated from motor imagery and motor imagery restraint.

The current study provides further support for the task-specific inhibitory mechanism of the SMC during motor imagery by dissociating between motor inhibition and motor imagery restraint. Our results show that motor imagery restraint specifically involves PPC. Previously, the PPC has been shown to activate when stopping from performing pre-planned movements (Garavan et al., 1999; Watanabe et al., 2002; Cavina-Pratesi et al., 2006) and inhibit volitional movements when electrically stimulated (Desmurget et al., 2018). However, in these studies, the prepotent behavior and the restraint were not experimentally separated, and it is unknown whether the inhibition occurred at the level of the motor output or the motor plan. Similarly, a recent electroencephalography GO/NO-GO study showed a significant parietal involvement during both motor execution and inhibition (Galdo-Alvarez et al., 2016). The current work experimentally separated the motor inhibition and restraint, where the participants were aware of the inherent requirement to inhibit any overt movements. Thus, the results showcase a correlation between the PPC BOLD activation specifically with motor imagery restraint.

The ratio of the GO and NO-GO trials was kept in favor of the former to induce an environment that strategically benefits the GO response (Wessel, 2018). The uneven ratio increases the likelihood of the response being planned, thereby encouraging the inhibitory responses upon the NO-GO cue. The task-specific inhibition of the SMC, along with the previous work showing motor imagery increasing corticospinal excitability (Fadiga et al., 1999; Grosprêtre et al., 2016) and somatotopic cortical activations (Lotze et al., 1999; Buccino et al., 2001; Ehrsson et al., 2003; Solodkin et al., 2004; Guillot and Collet, 2008), and congruent patterns of sub-threshold EMG activity during imagination of specific movements (Gandevia et al., 1997; Hashimoto and Rothwell, 1999), provides a level of confidence that the motor plans were actually planned, then were restrained upon the NO-GO cue. Importantly, there was a significant decrease in the BOLD activity below baseline in the same sub-regions of the SMC and the M1 that were activating during motor imagery that coincided at the same time as the PPC BOLD activation. The G-causal relationships from the PPC towards the SMC and the M1 are also consistent with the notion that the PPC inhibits the SMC and the M1 to inhibit motor plans in general for motor imagery restraint.

The current study focused on the cortical inhibitory mechanism coordinated from the SMC and the PPC, which resulted in a limited field-of-view. Previous studies have shown that other regions that could not be imaged in this study may play a role during motor inhibition (Coxon et al., 2006; Mirabella et al., 2012, 2013; Mattia et al., 2013). Further investigation is required to determine how the other regions of the broader motor network may be implicated in the cortical inhibitory mechanisms observed in the current study.

## Conclusion

We distinguished the neural correlates underlying two inhibitory mechanisms, motor inhibition and motor imagery restraint. Specific sub-regions of the SMC and the M1 showed BOLD activation during the imagination of specific movements and the G-causal relationship was found from the SMC to the M1. General BOLD activation in the PPC was observed during motor imagery restraint, which coincided with a BOLD deactivation in the same regions of the SMC and the M1 that were activating during motor imagery. Furthermore, G-causal relationships were observed from the PPC to the SMC and the M1. These results suggest that the SMC inhibits motor outputs in a task-specific manner, while the PPC inhibits motor plans to restrain responses in general, providing evidence that motor imagery involves inhibition at the level of motor output in a task-specific manner.

## Data Availability Statement

The datasets generated for this study are available on request to the corresponding author.

## Ethics Statement

The studies involving human participants were reviewed and approved by The University of Melbourne Human Ethics Committee; Ethics ID: 1340926.1. The patients/participants provided their written informed consent to participate in this study.

## Author Contributions

PY designed the experiments, performed the experiments, performed the analyses and wrote the manuscript. MH, SJ, NO, TO, SR, GR, AB, BM, and YW provided comments and made edits to the manuscript. BM and YW are joint last authors.

## Conflict of Interest

The authors declare that the research was conducted in the absence of any commercial or financial relationships that could be construed as a potential conflict of interest.
