# Supplementary material for: Distinct Neural Correlates Underlie Inhibitory Mechanisms of Motor Inhibition and Motor Imagery Restraint
Source: Front Behav Neurosci. 2020 Jun 3;14:77. doi: 10.3389/fnbeh.2020.00077 (PMC7289151; doi:10.3389/fnbeh.2020.00077)
Supplement: Supplementary file 1 [file Table_1.DOCX]

**Tables**

*“Distinct neural correlates underlie two inhibitory mechanisms of motor inhibition and motor cancellation”*

Yoo et al., 2019

| **Table 1.** Volume of activation during planning, imagination and restraint of motor imagery | | | | | | | | | |
| --- | --- | --- | --- | --- | --- | --- | --- | --- | --- |
|  | **Prompt: Average voxel volume ± standard error of BOLD activation at the individual level (mm^2^)** | | | | | | | | |
|  |  | **Imagined movement conditions** | | | | | | |  |
| **Regions** |  | **LA** |  | **RA** |  | **WF** |  | **LB** |  |
| **Bilateral PPC** |  | 6022±1375 |  | 5723±1219 |  | 6337±1180 |  | 6635±11880 |  |
| **Bilateral SMC** |  | 1516±445 |  | 1299±406 |  | 1553±466 |  | 1655±457 |  |
|  | **GO: Average voxel volume ± standard error of BOLD activation at the individual level (mm^2^)** | | | | | | | | |
|  |  | **Imagined movement conditions** | | | | | | |  |
| **Regions** |  | **LA** |  | **RA** |  | **WF** |  | **LB** |  |
| **Bilateral PPC** |  | 4503±1089 |  | 2602±944 |  | 2441±755 |  | 2191±863 |  |
| **Bilateral SMC** |  | 1179±292 |  | 640±212 |  | 831±269 |  | 550±204 |  |
| **Bilateral M1** |  | 555±199 |  | 257±87 |  | 409±179 |  | 302±32 |  |
|  | **NO-GO: Average voxel volume ± standard error of BOLD activation at the individual level (mm^2^)** | | | | | | | | |
| **Regions** |  | **Imagined movement conditions** | | | | | | |  |
| **Bilateral PPC** |  | 5715±688 | | | | | | |  |
| LA: left ankle; RA: right ankle; WF: walk forward; LB: lean back | | | | | | | | | |

| **Table 2.** Condition-specific, trial and time-point mean BOLD %∆*S* during ON and OFF-trials averaged across participants | | | | | | | | | | | | | |
| --- | --- | --- | --- | --- | --- | --- | --- | --- | --- | --- | --- | --- | --- |
|  | **Average %∆*S* ± standard deviation across participants %** | | | | | | | | | | | | |
|  |  | **Imagined movement conditions** | | | | | | | | | | |  |
| **Regions/**  **Condition** |  | **LA** | |  | **RA** | |  | **WF** | |  | **LB** | |  |
|  |  | **ON** | **OFF** |  | **ON** | **OFF** |  | **ON** | **OFF** |  | **ON** | **OFF** |  |
| **PPC/NO-GO** |  | 1.8±1.4 | 1.9±0.7 |  | 2.1±1.3 | 1.8±0.8 |  | 1.9±0.9 | 1.9±0.8 |  | 1.7±1.3 | 1.9±0.6 |  |
| **SMC/GO** |  | 2.7±1.0 | 1.8±0.9 |  | 2.9±1.6 | 2.0±1.4 |  | 2.6±1.6 | 1.4±1.3 |  | 3.4±2.3 | 1.7±1.3 |  |
| **M1/GO** |  | 2.4±1.5 | 0.8±1.1 |  | 1.7±1.2 | 0.7±1.1 |  | 2.3±1.5 | 0.5±0.9 |  | 2.8±1.5 | 0.7±1.0 |  |
| LA: left ankle; RA: right ankle; WF: walk forward; LB: lean back | | | | | | | | | | | | | |
